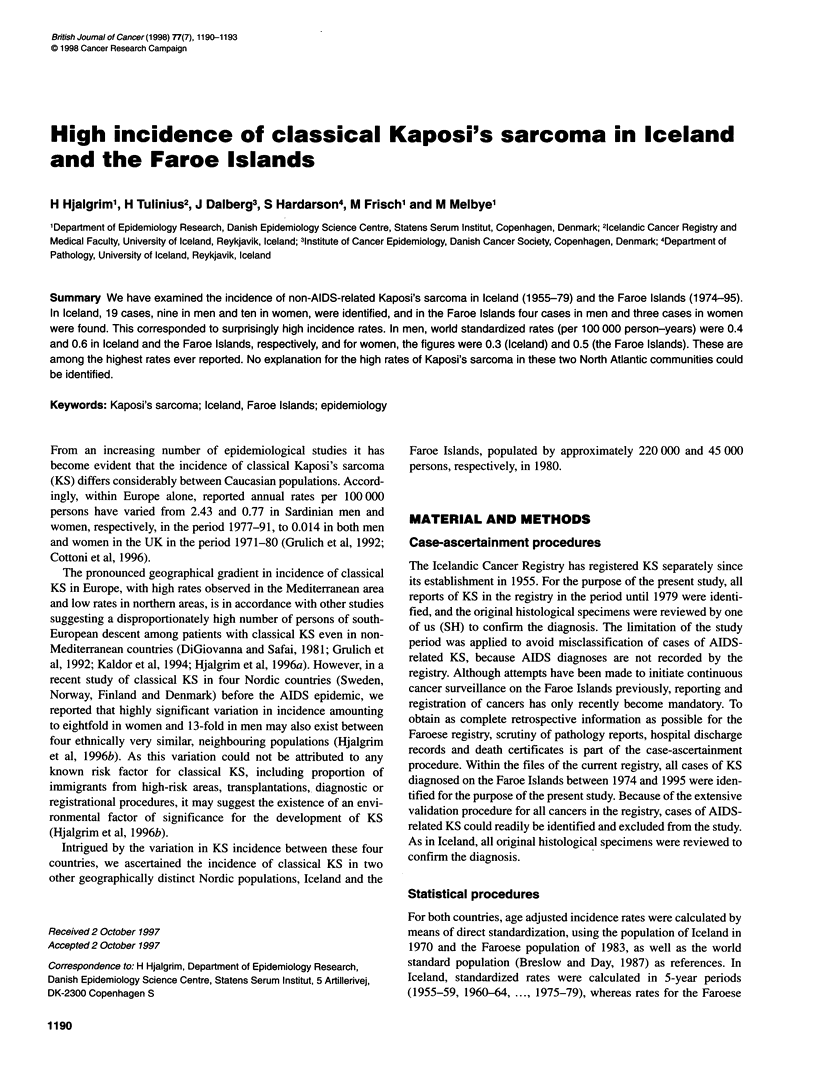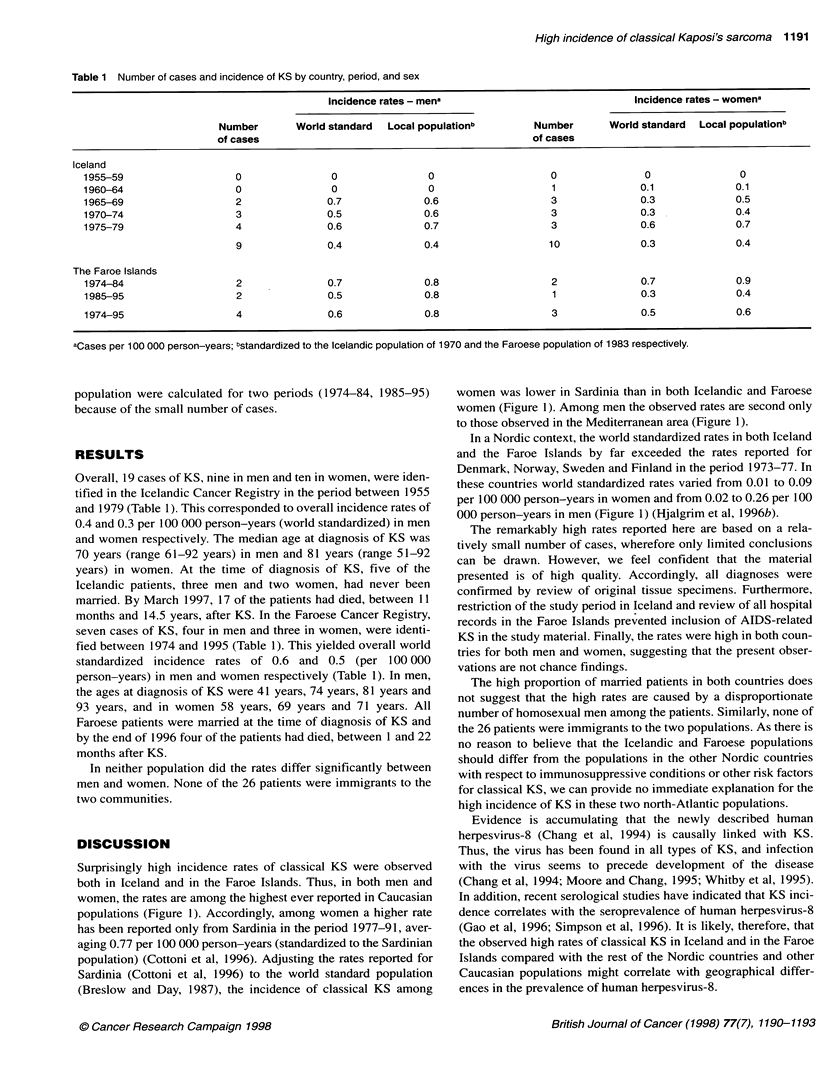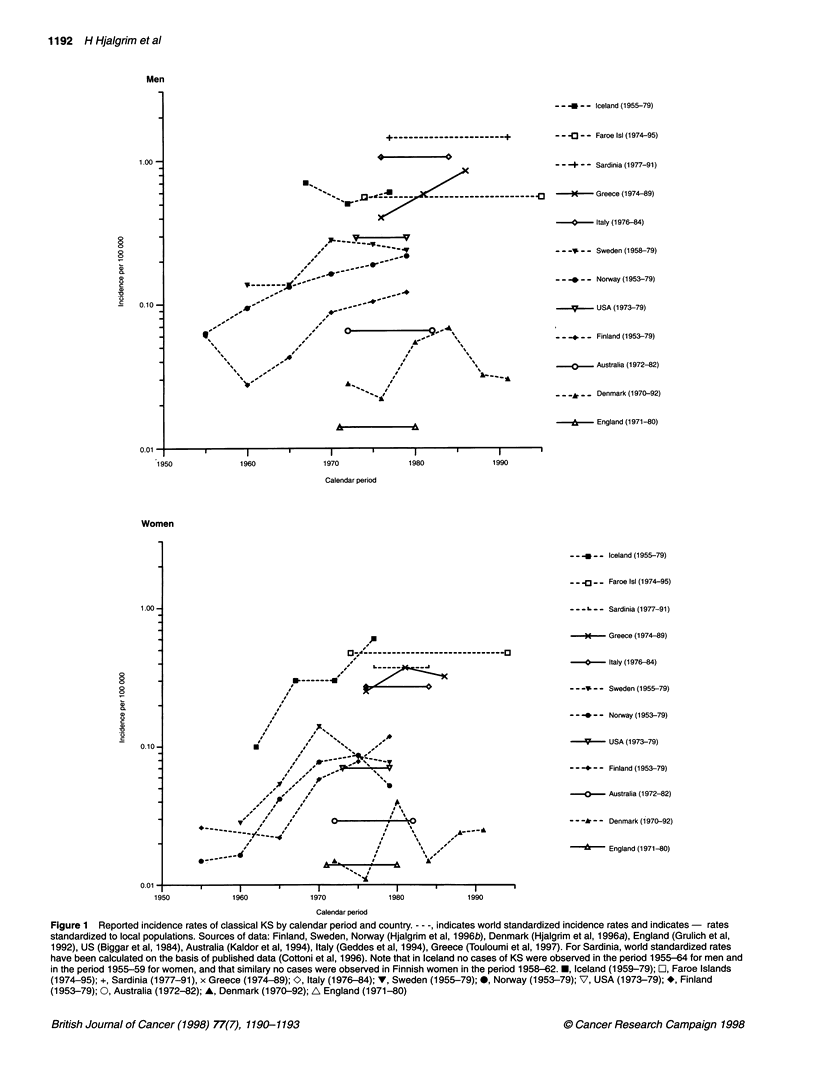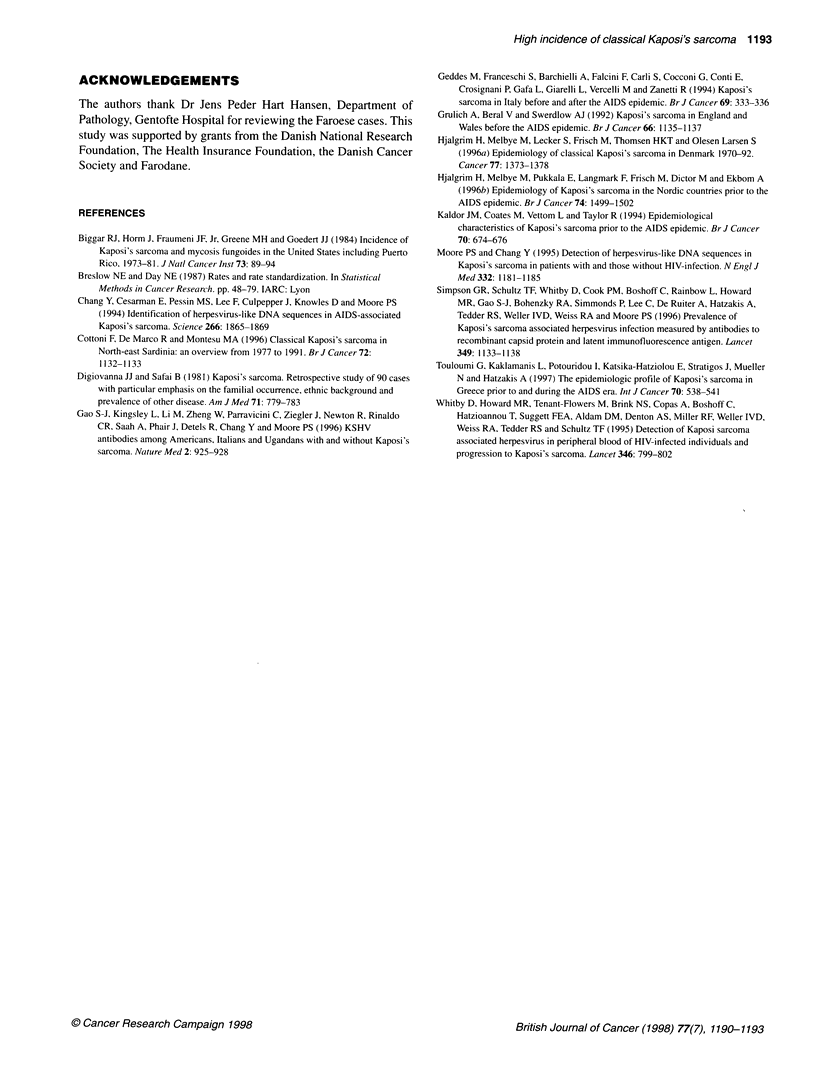# High incidence of classical Kaposi's sarcoma in Iceland and the Faroe Islands.

**DOI:** 10.1038/bjc.1998.198

**Published:** 1998-04

**Authors:** H. Hjalgrim, H. Tulinius, J. Dalberg, S. Hardarson, M. Frisch, M. Melbye

**Affiliations:** Department of Epidemiology Research, Danish Epidemiology Science Centre, Statens Serum Institut, Copenhagen.

## Abstract

We have examined the incidence of non-AIDS-related Kaposi's sarcoma in Iceland (1955-79) and the Faroe Islands (1974-95). In Iceland, 19 cases, nine in men and ten in women, were identified, and in the Faroe Islands four cases in men and three cases in women were found. This corresponded to surprisingly high incidence rates. In men, world standardized rates (per 100000 person-years) were 0.4 and 0.6 in Iceland and the Faroe Islands, respectively, and for women, the figures were 0.3 (Iceland) and 0.5 (the Faroe Islands). These are among the highest rates ever reported. No explanation for the high rates of Kaposi's sarcoma in these two North Atlantic communities could be identified.


British Journal of Cancer (1998) 77(7), 1190–1193
© 1998 Cancer Research Campaign

### High incidence of classical Kaposi's sarcoma in Iceland and the Faroe Islands

**H Hjalgrim[1], H Tulinius[2], J Dalberg[3], S Hardarson[4], M Frisch[1] and M Melbye[1]**

[1]Department of Epidemiology Research, Danish Epidemiology Science Centre, Statens Serum Institut, Copenhagen, Denmark; [2]Icelandic Cancer Registry and Medical Faculty, University of Iceland, Reykjavik, Iceland; [3]Institute of Cancer Epidemiology, Danish Cancer Society, Copenhagen, Denmark; [4]Department of Pathology, University of Iceland, Reykjavik, Iceland

**Summary** We have examined the incidence of non-AIDS-related Kaposi's sarcoma in Iceland (1955–79) and the Faroe Islands (1974–95). In Iceland, 19 cases, nine in men and ten in women, were identified, and in the Faroe Islands four cases in men and three cases in women were found. This corresponded to surprisingly high incidence rates. In men, world standardized rates (per 100 000 person–years) were 0.4 and 0.6 in Iceland and the Faroe Islands, respectively, and for women, the figures were 0.3 (Iceland) and 0.5 (the Faroe Islands). These are among the highest rates ever reported. No explanation for the high rates of Kaposi's sarcoma in these two North Atlantic communities could be identified.

**Keywords:** Kaposi's sarcoma; Iceland, Faroe Islands; epidemiology

From an increasing number of epidemiological studies it has become evident that the incidence of classical Kaposi's sarcoma (KS) differs considerably between Caucasian populations. Accordingly, within Europe alone, reported annual rates per 100 000 persons have varied from 2.43 and 0.77 in Sardinian men and women, respectively, in the period 1977–91, to 0.014 in both men and women in the UK in the period 1971–80 (Grulich et al, 1992; Cottoni et al, 1996).

The pronounced geographical gradient in incidence of classical KS in Europe, with high rates observed in the Mediterranean area and low rates in northern areas, is in accordance with other studies suggesting a disproportionately high number of persons of south-European descent among patients with classical KS even in non-Mediterranean countries (DiGiovanna and Safai, 1981; Grulich et al, 1992; Kaldor et al, 1994; Hjalgrim et al, 1996*a*). However, in a recent study of classical KS in four Nordic countries (Sweden, Norway, Finland and Denmark) before the AIDS epidemic, we reported that highly significant variation in incidence amounting to eightfold in women and 13-fold in men may also exist between four ethnically very similar, neighbouring populations (Hjalgrim et al, 1996*b*). As this variation could not be attributed to any known risk factor for classical KS, including proportion of immigrants from high-risk areas, transplantations, diagnostic or registrational procedures, it may suggest the existence of an environmental factor of significance for the development of KS (Hjalgrim et al, 1996*b*).

Intrigued by the variation in KS incidence between these four countries, we ascertained the incidence of classical KS in two other geographically distinct Nordic populations, Iceland and the Faroe Islands, populated by approximately 220 000 and 45 000 persons, respectively, in 1980.

*Received 2 October 1997*
*Accepted 2 October 1997*

*Correspondence to:* H Hjalgrim, Department of Epidemiology Research, Danish Epidemiology Science Centre, Statens Serum Institut, 5 Artillerivej, DK-2300 Copenhagen S

### MATERIAL AND METHODS

### Case-ascertainment procedures

The Icelandic Cancer Registry has registered KS separately since its establishment in 1955. For the purpose of the present study, all reports of KS in the registry in the period until 1979 were identified, and the original histological specimens were reviewed by one of us (SH) to confirm the diagnosis. The limitation of the study period was applied to avoid misclassification of cases of AIDS-related KS, because AIDS diagnoses are not recorded by the registry. Although attempts have been made to initiate continuous cancer surveillance on the Faroe Islands previously, reporting and registration of cancers has only recently become mandatory. To obtain as complete retrospective information as possible for the Faroese registry, scrutiny of pathology reports, hospital discharge records and death certificates is part of the case-ascertainment procedure. Within the files of the current registry, all cases of KS diagnosed on the Faroe Islands between 1974 and 1995 were identified for the purpose of the present study. Because of the extensive validation procedure for all cancers in the registry, cases of AIDS-related KS could readily be identified and excluded from the study. As in Iceland, all original histological specimens were reviewed to confirm the diagnosis.

### Statistical procedures

For both countries, age adjusted incidence rates were calculated by means of direct standardization, using the population of Iceland in 1970 and the Faroese population of 1983, as well as the world standard population (Breslow and Day, 1987) as references. In Iceland, standardized rates were calculated in 5-year periods (1955–59, 1960–64, ..., 1975–79), whereas rates for the Faroese

**Table 1** Number of cases and incidence of KS by country, period, and sex

| | Number of cases | Incidence rates – men[a] | | Number of cases | Incidence rates – women[a] | |
|---|---|---|---|---|---|---|
| | | World standard | Local population[b] | | World standard | Local population[b] |
| Iceland | | | | | | |
| 1955–59 | 0 | 0 | 0 | 0 | 0 | 0 |
| 1960–64 | 0 | 0 | 0 | 1 | 0.1 | 0.1 |
| 1965–69 | 2 | 0.7 | 0.6 | 3 | 0.3 | 0.5 |
| 1970–74 | 3 | 0.5 | 0.6 | 3 | 0.3 | 0.4 |
| 1975–79 | 4 | 0.6 | 0.7 | 3 | 0.6 | 0.7 |
| | 9 | 0.4 | 0.4 | 10 | 0.3 | 0.4 |
| The Faroe Islands | | | | | | |
| 1974–84 | 2 | 0.7 | 0.8 | 2 | 0.7 | 0.9 |
| 1985–95 | 2 | 0.5 | 0.8 | 1 | 0.3 | 0.4 |
| 1974–95 | 4 | 0.6 | 0.8 | 3 | 0.5 | 0.6 |

[a]Cases per 100 000 person–years; [b]standardized to the Icelandic population of 1970 and the Faroese population of 1983 respectively.

population were calculated for two periods (1974–84, 1985–95) because of the small number of cases.

### RESULTS

Overall, 19 cases of KS, nine in men and ten in women, were identified in the Icelandic Cancer Registry in the period between 1955 and 1979 (Table 1). This corresponded to overall incidence rates of 0.4 and 0.3 per 100 000 person–years (world standardized) in men and women respectively. The median age at diagnosis of KS was 70 years (range 61–92 years) in men and 81 years (range 51–92 years) in women. At the time of diagnosis of KS, five of the Icelandic patients, three men and two women, had never been married. By March 1997, 17 of the patients had died, between 11 months and 14.5 years, after KS. In the Faroese Cancer Registry, seven cases of KS, four in men and three in women, were identified between 1974 and 1995 (Table 1). This yielded overall world standardized incidence rates of 0.6 and 0.5 (per 100 000 person–years) in men and women respectively (Table 1). In men, the ages at diagnosis of KS were 41 years, 74 years, 81 years and 93 years, and in women 58 years, 69 years and 71 years. All Faroese patients were married at the time of diagnosis of KS and by the end of 1996 four of the patients had died, between 1 and 22 months after KS.

In neither population did the rates differ significantly between men and women. None of the 26 patients were immigrants to the two communities.

### DISCUSSION

Surprisingly high incidence rates of classical KS were observed both in Iceland and in the Faroe Islands. Thus, in both men and women, the rates are among the highest ever reported in Caucasian populations (Figure 1). Accordingly, among women a higher rate has been reported only from Sardinia in the period 1977–91, averaging 0.77 per 100 000 person–years (standardized to the Sardinian population) (Cottoni et al, 1996). Adjusting the rates reported for Sardinia (Cottoni et al, 1996) to the world standard population (Breslow and Day, 1987), the incidence of classical KS among women was lower in Sardinia than in both Icelandic and Faroese women (Figure 1). Among men the observed rates are second only to those observed in the Mediterranean area (Figure 1).

In a Nordic context, the world standardized rates in both Iceland and the Faroe Islands by far exceeded the rates reported for Denmark, Norway, Sweden and Finland in the period 1973–77. In these countries world standardized rates varied from 0.01 to 0.09 per 100 000 person–years in women and from 0.02 to 0.26 per 100 000 person–years in men (Figure 1) (Hjalgrim et al, 1996*b*).

The remarkably high rates reported here are based on a relatively small number of cases, wherefore only limited conclusions can be drawn. However, we feel confident that the material presented is of high quality. Accordingly, all diagnoses were confirmed by review of original tissue specimens. Furthermore, restriction of the study period in Iceland and review of all hospital records in the Faroe Islands prevented inclusion of AIDS-related KS in the study material. Finally, the rates were high in both countries for both men and women, suggesting that the present observations are not chance findings.

The high proportion of married patients in both countries does not suggest that the high rates are caused by a disproportionate number of homosexual men among the patients. Similarly, none of the 26 patients were immigrants to the two populations. As there is no reason to believe that the Icelandic and Faroese populations should differ from the populations in the other Nordic countries with respect to immunosuppressive conditions or other risk factors for classical KS, we can provide no immediate explanation for the high incidence of KS in these two north-Atlantic populations.

Evidence is accumulating that the newly described human herpesvirus-8 (Chang et al, 1994) is causally linked with KS. Thus, the virus has been found in all types of KS, and infection with the virus seems to precede development of the disease (Chang et al, 1994; Moore and Chang, 1995; Whitby et al, 1995). In addition, recent serological studies have indicated that KS incidence correlates with the seroprevalence of human herpesvirus-8 (Gao et al, 1996; Simpson et al, 1996). It is likely, therefore, that the observed high rates of classical KS in Iceland and in the Faroe Islands compared with the rest of the Nordic countries and other Caucasian populations might correlate with geographical differences in the prevalence of human herpesvirus-8.

© Cancer Research Campaign 1998

**Figure 1** Reported incidence rates of classical KS by calendar period and country. - - -, indicates world standardized incidence rates and indicates — rates standardized to local populations. Sources of data: Finland, Sweden, Norway (Hjalgrim et al, 1996*b*), Denmark (Hjalgrim et al, 1996*a*), England (Grulich et al, 1992), US (Biggar et al, 1984), Australia (Kaldor et al, 1994), Italy (Geddes et al, 1994), Greece (Touloumi et al, 1997). For Sardinia, world standardized rates have been calculated on the basis of published data (Cottoni et al, 1996). Note that in Iceland no cases of KS were observed in the period 1955–64 for men and in the period 1955–59 for women, and that similary no cases were observed in Finnish women in the period 1958–62. ■, Iceland (1959–79); □, Faroe Islands (1974–95); +, Sardinia (1977–91), × Greece (1974–89); ◇, Italy (1976–84); ▼, Sweden (1955–79); ●, Norway (1953–79); ▽, USA (1973–79); ◆, Finland (1953–79); ○, Australia (1972–82); ▲, Denmark (1970–92); △ England (1971–80)

 *© Cancer Research Campaign 1998*

### ACKNOWLEDGEMENTS

The authors thank Dr Jens Peder Hart Hansen, Department of Pathology, Gentofte Hospital for reviewing the Faroese cases. This study was supported by grants from the Danish National Research Foundation, The Health Insurance Foundation, the Danish Cancer Society and Farodane.

### REFERENCES

Biggar RJ, Horm J, Fraumeni JF, Jr, Greene MH and Goedert JJ (1984) Incidence of Kaposi's sarcoma and mycosis fungoides in the United States including Puerto Rico, 1973–81. *J Natl Cancer Inst* **73**: 89–94

Breslow NE and Day NE (1987) Rates and rate standardization. In *Statistical Methods in Cancer Research*. pp. 48–79. IARC: Lyon

Chang Y, Cesarman E, Pessin MS, Lee F, Culpepper J, Knowles D and Moore PS (1994) Identification of herpesvirus-like DNA sequences in AIDS-associated Kaposi's sarcoma. *Science* **266**: 1865–1869

Cottoni F, De Marco R and Montesu MA (1996) Classical Kaposi's sarcoma in North-east Sardinia: an overview from 1977 to 1991. *Br J Cancer* **72**: 1132–1133

Digiovanna JJ and Safai B (1981) Kaposi's sarcoma. Retrospective study of 90 cases with particular emphasis on the familial occurrence, ethnic background and prevalence of other disease. *Am J Med* **71**: 779–783

Gao S-J, Kingsley L, Li M, Zheng W, Parravicini C, Ziegler J, Newton R, Rinaldo CR, Saah A, Phair J, Detels R, Chang Y and Moore PS (1996) KSHV antibodies among Americans, Italians and Ugandans with and without Kaposi's sarcoma. *Nature Med* **2**: 925–928

Geddes M, Franceschi S, Barchielli A, Falcini F, Carli S, Cocconi G, Conti E, Crosignani P, Gafa L, Giarelli L, Vercelli M and Zanetti R (1994) Kaposi's sarcoma in Italy before and after the AIDS epidemic. *Br J Cancer* **69**: 333–336

Grulich A, Beral V and Swerdlow AJ (1992) Kaposi's sarcoma in England and Wales before the AIDS epidemic. *Br J Cancer* **66**: 1135–1137

Hjalgrim H, Melbye M, Lecker S, Frisch M, Thomsen HKT and Olesen Larsen S (1996*a*) Epidemiology of classical Kaposi's sarcoma in Denmark 1970–92. *Cancer* **77**: 1373–1378

Hjalgrim H, Melbye M, Pukkala E, Langmark F, Frisch M, Dictor M and Ekbom A (1996*b*) Epidemiology of Kaposi's sarcoma in the Nordic countries prior to the AIDS epidemic. *Br J Cancer* **74**: 1499–1502

Kaldor JM, Coates M, Vettom L and Taylor R (1994) Epidemiological characteristics of Kaposi's sarcoma prior to the AIDS epidemic. *Br J Cancer* **70**: 674–676

Moore PS and Chang Y (1995) Detection of herpesvirus-like DNA sequences in Kaposi's sarcoma in patients with and those without HIV-infection. *N Engl J Med* **332**: 1181–1185

Simpson GR, Schultz TF, Whitby D, Cook PM, Boshoff C, Rainbow L, Howard MR, Gao S-J, Bohenzky RA, Simmonds P, Lee C, De Ruiter A, Hatzakis A, Tedder RS, Weller IVD, Weiss RA and Moore PS (1996) Prevalence of Kaposi's sarcoma associated herpesvirus infection measured by antibodies to recombinant capsid protein and latent immunofluorescence antigen. *Lancet* **349**: 1133–1138

Touloumi G, Kaklamanis L, Potouridou I, Katsika-Hatziolou E, Stratigos J, Mueller N and Hatzakis A (1997) The epidemiologic profile of Kaposi's sarcoma in Greece prior to and during the AIDS era. *Int J Cancer* **70**: 538–541

Whitby D, Howard MR, Tenant-Flowers M, Brink NS, Copas A, Boshoff C, Hatzioannou T, Suggett FEA, Aldam DM, Denton AS, Miller RF, Weller IVD, Weiss RA, Tedder RS and Schultz TF (1995) Detection of Kaposi sarcoma associated herpesvirus in peripheral blood of HIV-infected individuals and progression to Kaposi's sarcoma. *Lancet* **346**: 799–802

© Cancer Research Campaign 1998